# Effects of G-Quadruplex-Binding Plant Secondary Metabolites on *c-MYC* Expression

**DOI:** 10.3390/ijms23169209

**Published:** 2022-08-16

**Authors:** Roman G. Zenkov, Kirill I. Kirsanov, Anna M. Ogloblina, Olga A. Vlasova, Denis S. Naberezhnov, Natalia Y. Karpechenko, Timur I. Fetisov, Ekaterina A. Lesovaya, Gennady A. Belitsky, Nina G. Dolinnaya, Marianna G. Yakubovskaya

**Affiliations:** 1 N. N. Blokhin Russian Cancer Research Center, 24 Kashirskoe Shosse, Moscow 115478, Russia; 2 Institute of Medicine, RUDN University, 6 Miklukho-Maklaya St., Moscow 117198, Russia; 3 Department of Medicinal Chemistry and Toxicology, Pirogov Russian National Research Medical University, 1 Ostrovityanova St., Moscow 117997, Russia; 4 Department of Oncology, I.P. Pavlov Ryazan State Medical University, 9 Vysokovoltnaya St., Ryazan 390026, Russia; 5 Department of Chemistry, Lomonosov Moscow State University, Leninskie Gory 1, Moscow 119991, Russia

**Keywords:** G-quadruplex, G4-binding ligands, plant secondary metabolites, luciferase reporter assay, *c-MYC* promoter, fluorescent indicator displacement, gene expression regulation

## Abstract

Guanine-rich DNA sequences tending to adopt noncanonical G-quadruplex (G4) structures are over-represented in promoter regions of oncogenes. Ligands recognizing G4 were shown to stabilize these DNA structures and drive their formation regulating expression of corresponding genes. We studied the interaction of several plant secondary metabolites (PSMs) with G4s and their effects on gene expression in a cellular context. The binding of PSMs with G4s formed by the sequences of well-studied oncogene promoters and telomeric repeats was evaluated using a fluorescent indicator displacement assay. *c-MYC* G4 folding topology and thermal stability, as well as the PMS influence on these parameters, were demonstrated by UV-spectroscopy and circular dichroism. The effects of promising PSMs on *c-MYC* expression were assessed using luciferase reporter assay and qPR-PCR in cancer and immortalized cultured cells. The ability of PMS to multi-targeting cell signaling pathways was analyzed by the pathway-focused gene expression profiling with qRT-PCR. The multi-target activity of a number of PSMs was demonstrated by their interaction with a set of G4s mimicking those formed in the human genome. We have shown a direct G4-mediated down regulation of *c-MYC* expression by sanguinarine, quercetin, kaempferol, and thymoquinone; these effects being modulated by PSM’s indirect influence via cell signaling pathways.

## 1. Introduction

Tandem guanine repeats capable of folding into G-quadruplexes (G4s), alternative DNA structures, are known as G4 motifs. G4s are formed by stacking of G-tetrads comprised of four guanine bases held together in a square planar arrangement by Hoogsteen hydrogen bonds [1,2]. There is abundant evidence that the formation of G4s is associated with the regulation of various cellular processes, including replication initiation [3], DNA recombination [4], telomere maintenance [5,6], DNA repair [7], mutagenesis [8], regulation of transcription [9,10] and others. Among them, the direct effects of G4s on gene expression are of greatest interest due to their possible application in cancer therapy [11]. In the eukaryotic genome, promoter regions are significantly enriched with G4 motifs, demonstrating their higher density compared to the average value in the genome [12]. G4 location in the genome also indicates an interplay between G4s and gene functions: the frequency of G4-forming sequences in the promoter regions of many oncogenes and genes involved in growth control is above the average value and in the promoters of tumor suppressor genes below average [11,12]. Many studies have revealed a variety of synthetic and natural small molecule ligands that recognize these noncanonical DNA structures and drive G4 folding to initiate oncogene silencing [13,14]. Most of these compounds have a planar aromatic ring system that can stabilize G4s. Several plant secondary metabolites (PSMs) have been shown to share this feature and bind G4s: fisetin, sanguinarine, thymoquinone, quercetin, and berberine [15,16,17,18,19].

The main goal of our study was to analyze the interaction of various PSMs, known for their diverse beneficial effects on human health, with a set of G4s potentially formed in the promoters of well-known human oncogenes. Next, we would like to analyze whether PSMs that bind to the G4 of oncogene promoter can influence gene expression in a cellular context and, finally, how this targeted effect is realized against the complex indirect effects of PSMs via cell signaling pathways.

We characterized the binding properties of 11 PSMs (sanguinarine, quercetin, epigallocatechin gallate, kaempferol, thymoquinone, curcumin, apigenin, coumarin, resveratrol, genistein (EGCG), and naringenin) to G4 structures adapted by the promoter regions of 6 known human oncogenes using the G4 fluorescent indicator displacement assay (G4-FID) [20]. Based on these data, we assessed the ability of 4 representative PSM ligands with optimal G4 binding properties (sanguinarine, quercetin, kaempferol, and thymoquinone) to regulate reporter gene transcription controlled by the *c-MYC* promoter. The G4 motif of the *c-MYC* promoter was chosen for our subsequent study as this gene product is the major oncogenic driver in cancer [13,21,22]. To evaluate the G4-mediated effect of the ligands on *c-MYC* expression, we used the model system elaborated by Siddiqui-Jain et al. [23,24]. Coupled expression of the luciferase reporter gene controlled by a well-studied G4-forming sequence originated from the nuclease hypersensitive element (NHE) III_1_ of the *c-MYC* promoter and by the same sequence but with a G to A substitution preventing G4 folding was measured to equalize interfering indirect influences and, thereby to reveal the targeted G4-mediated effect on the reporter gene expression. Next, we used qRT-PCR to estimate the total effects of PSMs on *c-MYC* expression in four cell lines using the Human Signal Transduction PathwayFinder RT^2^ Profiler PCR Array to analyze the effect of PSMs on the expression of key genes of different signaling pathways.

## 2. Results

### 2.1. Evaluation of PSM Binding to the Promoter G4s Using the G4-FID Assay

First, we examined the affinity of various PSMs for DNA oligonucleotides mimicking the G-rich promoter regions of the best-known oncogenes capable of folding into G4 structures (Table 1). Stabilization of these G4s has been previously proved to cause down-regulation of gene expression. As parallel folding is the main biologically relevant topology of the promoter G4s [25,26], the Tel oligonucleotide containing four human telomeric repeats (5′-GGGTTAGGGTTAGGGTTAGGGTTA) [27] was characterized for comparison. Unlike parallel promoter G4s, telomeric DNA folds into a G4 structure with an antiparallel and hybrid (3 + 1) arrangement of G-tracts [28].

**Figure 1:**
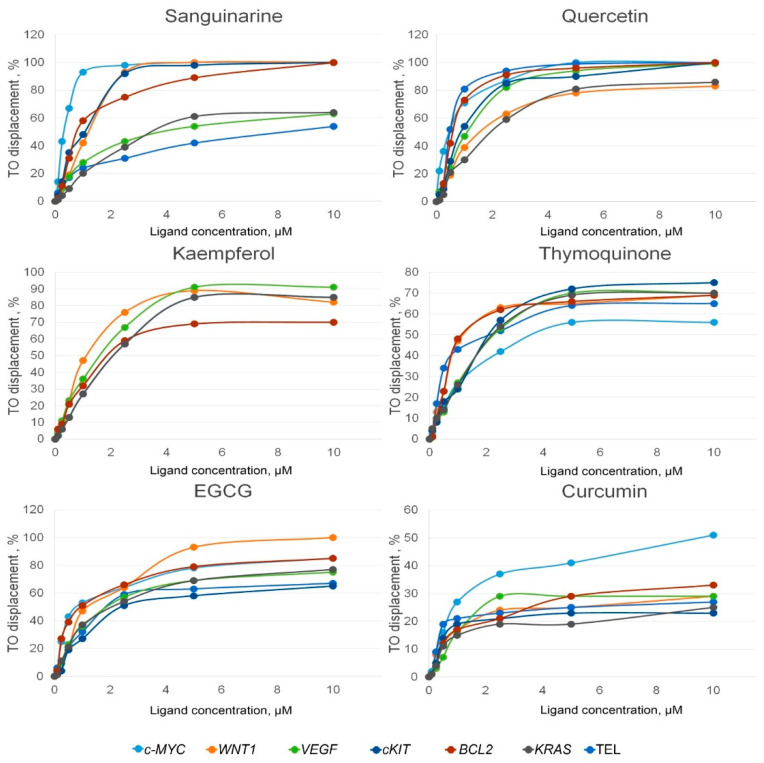
TO displacement from G4s formed by synthetic DNA oligonucleotides mimicking G-rich promoter regions of various oncogenes, depending on the concentration of added PSMs.

The PSM binding to G4s was evaluated by G4-FID assay [20,35,36]. This method is based on the loss of thiazole orange (TO) fluorescence upon competitive displacement from DNA by a putative ligand. The percentages of TO displacement from different G4s plotted against the concentration of the PSM are shown in Figure 1 and Figure S1.

Based on these curves, we estimated DC_50_ values for all PSM ligands and G4s used, as described by Monchaud et al. [35,36] (Table 2). Binding constants characterizing the affinity of PSMs were calculated using the values of PSM DC_50_ and the binding constant of TO [20,37,38].

We analyzed the G4 binding capacity of sanguinarine, quercetin, EGCG, kaempferol, thymoquinone, curcumin, apigenin, coumarin, resveratrol, genistein, and naringenin. However, only sanguinarine, quercetin, EGCG, kaempferol, thymoquinone, and curcumin showed a pronounced affinity for the G4 structures. The values of the binding constants depended both on the G4 topology (parallel or antiparallel) and on the type of ligand; however, the interaction of PSMs with different G4s revealed their multi-target ability (Figure 1 and Table 2).

Among all the studied ligands, sanguinarine demonstrated the highest affinity for the promoter parallel G4s with a binding constant ranging from 7.76 to 0.86 × 10^6^ M^−1^. But its affinity for telomeric antiparallel G4 (0.43 × 10^6^ M^−1^) was much lower. The binding efficiency of other PSMs to various G4s also differed to some extent, indicating their G4 structural selectivity. Thus, thymoquinone showed a 2.66 times higher affinity for the telomeric G4 compared to *c-MYC* G4, but its highest binding constant (2.87 × 10^6^ M^−1^) was observed for the *BCL2* quadruplex.

Curcumin showed poor binding only to the *c-MYC* promoter G4 (DC_50_ was 9.62 ± 0.70 µM and *K_b_* − 0.26 ± 0.02 × 10^6^ M^−1^). Its binding affinity for other quadruplex targets was insufficient to displace 50% of TO even at a concentration of 10 µM. Interaction with G4s was not found for apigenin, resveratrol, genistein and naringenin. Probably, their affinity for G4 targets is insufficient to displace TO, or they bind to different G4 sites that do not overlap with TO binding sites (Figure S2). None of these compounds fluoresces in the TO fluorescence wavelength range (Table S2 and Figure S3).

The binding affinity of the most promising PSMs, sanguinarine, quercetin, kaempferol, and thymoquinone, to the DNA duplex target, hairpin DNA, was also evaluated (Table 2). As can be seen, the PSM binding constant for the DNA duplex was significantly lower than for the *c-MYC* G4 promoter, which was selected for comparative analysis. Based on these data, the selectivity factors for each ligand were calculated as the ratio of the DC_50 DNA hairpin_ to the value of DC_50 *c-MYC*_; they were 9.63, 52.91, and 223.96 for sanguinarine, kaempferol, and quercetin, respectively; Thymoquinone exhibited absolute G4 selectivity, since it did not bind to duplex DNA at all. These data indicate that quercetin and thymoquinone interact most selectively with *c-MYC* G4 compared to other studied PSMs and allow discrimination between DNA quadruplex and duplex structures.

### 2.2. The Thermal Stability of c-MYC G4 Measured by UV and CD Spectroscopy Methods in the Presence of PSMs

**Figure 2:**
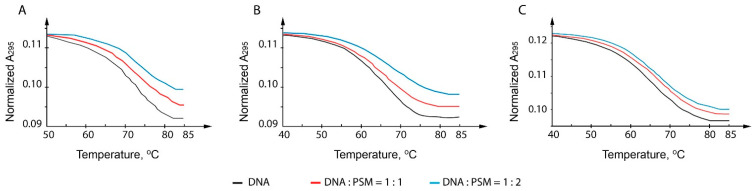
Melting profiles of *c-MYC* G4 alone (**A**–**C**) and in the presence of 1 or 2 equivalents of PSMs: sanguinarine (**A**,**B**) and quercetin (**C**). The temperature dependence of UV absorption was recorded at 295 nm in buffer A (**A**) or buffer B (**B**,**C**).

To confirm the G4 formation by the 26-nt *c-MYC* promoter fragment and to elucidate the quadruplex-stabilizing effects of the most promising PSMs, thermal melting curves recorded at 295 nm were characterized. The temperature dependence of UV absorbance at 295 nm is a G4 structure marker. Unlike the DNA duplex, whose melting is accompanied by a hyperchromic effect (typically at 260 nm), G4 melting at 295 nm causes a decrease in optical density, and this is a cooperative process. All measurements were performed in buffer solutions containing, along with the sodium ions (140 mM), 0.5 or 5.0 mM KCl. These deliberately low K^+^ concentrations, far from those in human cells (~150 mM KCl), were chosen to capture the entire conformation transition in the available temperature range. The unfolding profiles of *c-MYC* promoter oligonucleotide demonstrate a clear single G4-coil transition at 295 nm with the melting temperature (T_m_) values of 64 ± 0.3 °C at 0.5 mM KCl and 71.7 ± 0.3 °C at 5.0 mM KCl (Figure 2). These data confirm that the *c-MYC* promoter fragment used in our study folds into an extremely stable G4 even under potassium-depleted conditions. The addition of sanguinarine to the probe in buffer A (with 5.0 mM KCl) at a ratio of 1:1 or 1:2 (G4:ligand) resulted in a clear stabilization of G4 (Figure 2A). However, quantitative data were obtained only in 0.5 mM KCl-containing buffer B; one and two equivalents of sanguinarine caused an increase in the G4 T_m_ value by 1.5 and 3.5 ± 0.3 °C, respectively, compared with the free oligonucleotide (Figure 2B). Under the same conditions, one and two equivalents of quercetin stabilized the *c-MYC* quadruplex by 1.2 and 3.0 ± 0.3 °C, respectively (Figure 2C). The presence of one or two equivalents of kaempferol and thymoquinone did not significantly affect *c-MYC* G4 stability (data not shown).

**Figure 3:**
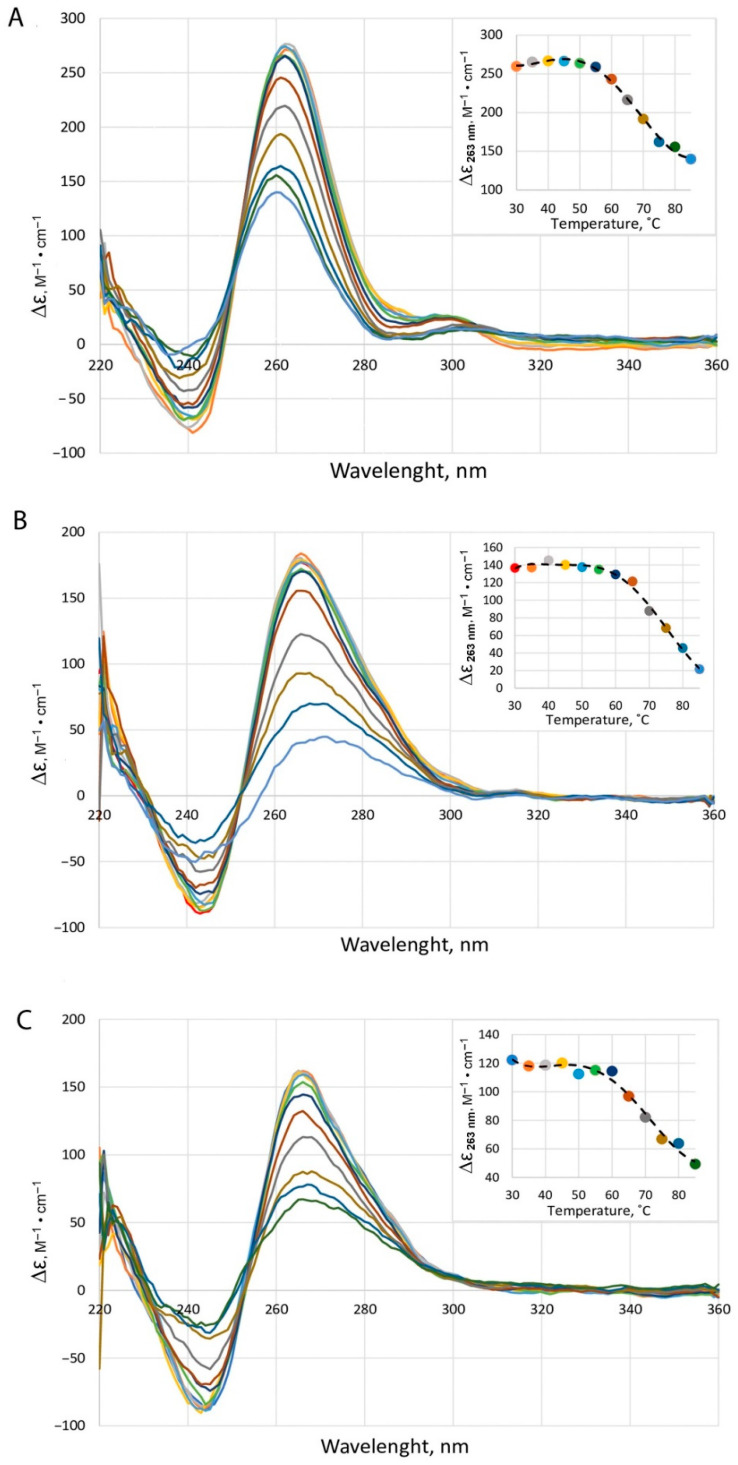
CD spectra and melting profiles of *c-MYC* G4 alone (~4 μM oligonucleotide strand concentration) (**A**) and in the presence of an equimolar DNA duplex and either sanguinarine (**B**) or quercetin (**C**) taken in a 1:2 ratio (G4:ligand). CD spectra were recorded in buffer B at different temperatures; the temperature was raised from 30 to 85 °C in 5 °C increments. (Insets) CD-monitored melting profiles at 263 nm.

To characterize the topology of *c-MYC* G4 and the effect of PSMs on G4 conformation, circular dichroism (CD) measurements were performed. CD spectroscopy was also used to test whether the addition of a dsDNA, which competes with *c-MYC* G4 for ligand binding, reduced the level of PSM-induced G4 stabilization. CD data recorded at different temperatures made it possible to obtain melting curves for all studied DNA samples (Figure 3, insets), which contain *c-MYC* G4 alone (Figure 3A) and the same quadruplex with the addition of an equimolar dsDNA and either sanguinarine (Figure 3B) or quercetin (Figure 3C) in a 1:2 ratio (G4:ligand). It was these PSM ligands that caused an increase in the T_m_ values of *c-MYC* G4 by 3–3.5 °C in a buffer solution containing 0.5 mM KCl compared to free *c-MYC* G4 (Figure 2). The CD spectrum of *c-MYC* G4 alone revealed a typical parallel G4 fold with a positive peak at 263 nm and a negative one at 240 nm. The addition of small molecule ligands in a 1:2 ratio (G4:ligand) and the equimolar dsDNA led to a broadening of the positive band and a shift of the peak maximum to 268 nm, which was due to the presence of a DNA duplex structure, since the characteristic positive CD band of B-DNA is located at 270–280 nm. However, the effect of dsDNA on the total CD spectrum is masked by an intense positive peak corresponding to parallel-stranded G4. Considering this, it can be concluded that PSM ligands cause only minor changes in the CD spectrum of *c-MYC* G4 itself, retaining all the typical signatures of parallel G4 topology (Figure 3 and Figure S4).

The CD melting curve for free *c-MYC* G4, determined at 263 nm with T_m_ of 65 ± 1.5 °C, agrees well with the temperature dependence of UV absorbance at 295 nm (T_m_ = 64 ± 0.3 °C). Importantly, CD melting profiles for *c-MYC* G4 in the presence of duplex and sanguinarine (Figure 3B) or quercetin (Figure 3C) show higher T_m_ values close to those for *c-MYC* G4 after treatment with these PSMs, but without DNA duplexes (Figure 2), thus confirming the preferential binding of these ligands to the parallel G4 structure over duplex targeting. The obtained CD data confirm the selectivity of PSM binding to *c-MYC* G4 estimated by FID assay, which makes it possible to distinguish between quadruplex and duplex DNA structures.

### 2.3. PSMs Down-Regulate the Promoter Activity of c-MYC by Targeting Its G4 Structure

**Figure 4:**
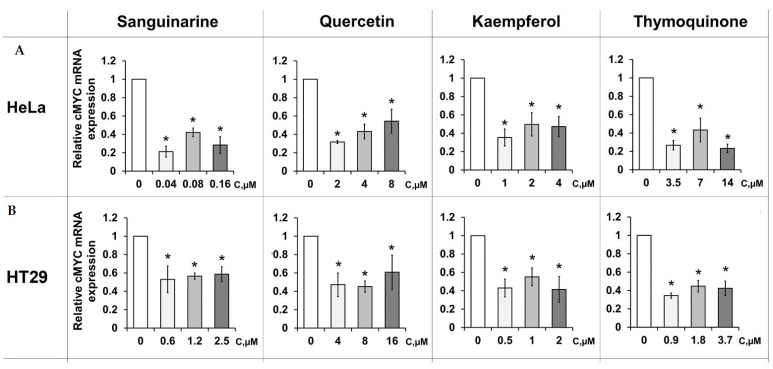
Effects of PSMs on *c-MYC*-controlled expression in HeLa (**A**) and HT29 (**B**) cells as assessed by luciferase reporter assay. All data are presented as M ± m. * Differences are statistically significant compared to control (*p* < 0.05).

Since sanguinarine, quercetin, kaempferol, and thymoquinone were found to be selective PSMs for targeting promoter G4s, their direct effect on *c-MYC* gene expression in a cellular context was evaluated using a luciferase reporter assay. To perform this analysis, two DNA fragments containing (i) wild-type *c-MYC* G4 motif and (ii) the same sequence but with one G→A nucleotide substitution preventing G4 formation were cloned into the upstream region of pGL3-Basic firefly luciferase promoterless vector. Both plasmids were transiently transfected into human cervical cancer (HeLa) and human colorectal cancer (HT29) cell lines, followed by treatment with PSMs. To examine whether the effect of PSMs was a consequence of *c-MYC* G4 targeting, we normalized firefly luciferase expression from the wild-type *c-MYC* promoter harboring the G4 motif relative to the expression from the altered *c-MYC* promoter, the sequence of which has a single G→A substitution. The known G4 stabilizer TMPyP4 (5,10,15,20-tetrakis-(N-methyl-4-pyridyl)porphyrin) was used as a positive control. This ligand at a concentration of 100 µM reduced the wild-type *c-MYC* promoter-linked luciferase expression by up to 28.6% compared to the untreated sample. Among the investigated PSMs in the HeLa cell line, sanguinarine turned out to be the most active compound, suppressing the luciferase expression up to 21.2% at a concentration of 0.04 µM (Figure 4).

Similar changes in the relative expression of the reporter vector were observed with thymoquinone treatment; this ligand, taken at a concentration of 14 µM, reduced the luciferase expression to 23.3%. Quercetin and kaempferol showed almost equal effects, reducing *c-MYC*-controlled expression by up to 31.7 and 35.4%, respectively. Luciferase assay in another cell line, HT29, revealed thymoquinone as the most active compound reducing relative luciferase activity to 34.4% at a concentration of 0.9 µM. Quercetin and kaempferol downregulated the signal to 45.1 and 41.4%, respectively. Finally, sanguinarine demonstrated the lowest effect—it reduced relative luciferase activity to 53% at a concentration of 0.6 µM. These results revealed the G4-mediated inhibiting effect of PSMs on *c-MYC* expression.

### 2.4. PSM-Dependent *c-MYC Gene* Expression in Cultured Cells

**Figure 5:**
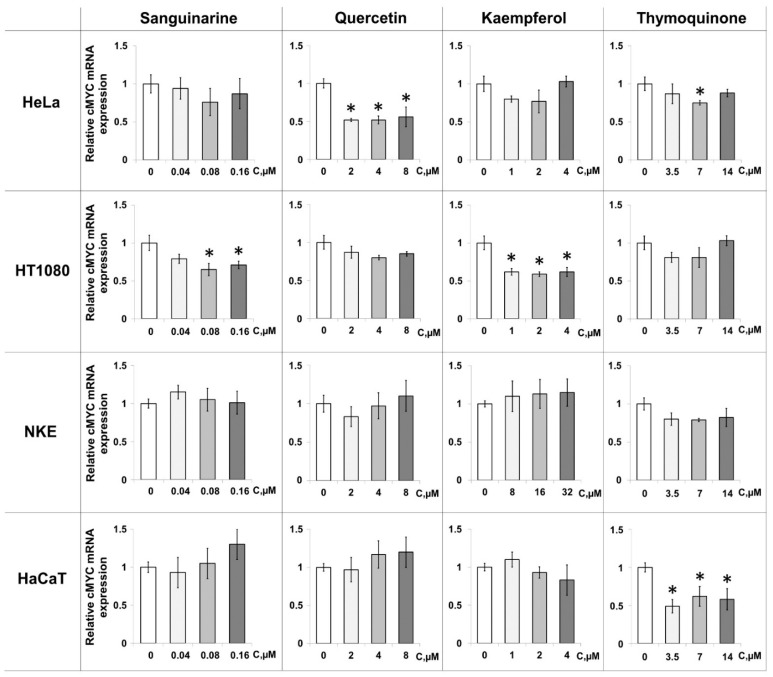
Effects of PSMs on *c-MYC* expression determined by RT-PCR in cancer (HeLa and HT1080) and immortalized (HaCaT and NKE) cell lines. All data are presented as M ± m. * Differences are statistically significant compared to control (*p* < 0.05).

To further explore the total effects of PSMs in a cellular context, we evaluated their ability to regulate *c-MYC* transcription using qRT-PCR analysis. The effects of sanguinarine, quercetin, kaempferol, and thymoquinone were analyzed by monitoring *c-MYC* mRNA expression levels in cancer and immortalized cell lines. Cells were treated with three different concentrations of ligands for 48 h. The maximum nontoxic concentrations of the PSMs were determined by the MTT assay; the other two doses were 2 and 4 times lower, respectively (Table S1). To normalize the obtained data, the expression levels of RPL27, RPLP0, and GPI genes that do not contain G4 motifs in their promoter regions were assessed in the same cells after the treatment with PSMs. Quercetin, kaempferol, and thymoquinone reduced *c-MYC* expression in HeLa and HT1080 malignant cell lines. However, the intensity of the effects varied depending on the cell type (Figure 5).

Our data showed that quercetin was more active in HeLa cells: their treatment with 4 and 2 μM PSM resulted in a 2-fold decrease in *c-MYC* transcription compared to the control level. A significant effect was also observed for thymoquinone, with transcription efficiency reduced to 75% of the control level after the treatment with 7 μM plant metabolite. Kaempferol at a dose of 2 μM significantly reduced *c-MYC* transcription in malignant HT1080 cells (up to 59%) and less effectively in HeLa (up to 77%). Surprisingly, the most effective binder, sanguinarine, did not reduce *c-MYC* expression in HeLa cells. However, treatment of another cancer cell line, HT1080, with both 0.16 and 0.08 μM sanguinarine reduced the level of *c-MYC* transcription to 71 and 65%, respectively.

PSMs did not significantly reduce *c-MYC* expression in immortalized (quasi-normal) HaCaT and NKE cells, with one exception: in the HaCaT cell line, thymoquinone markedly inhibited *c-MYC* transcription at all concentrations used.

Some of the discrepancies between the results of PMS influence on *c-MYC* expression obtained by the luciferase assay and the qRT-PCR analysis may be due to the confounding indirect effects of PMSs on cell signaling; PSM ligands are known to be multi-targeted compounds and their binding to G4s of different oncogenes was demonstrated in this work. In this regard, to analyze the influence of PSMs on different signal pathways, we performed a PCR array using the RT^2^ Profiler^TM^ PCR Array Human Signal Transduction Pathway Finder (QIAGEN, Germany) (Table S3). It includes primers for 84 target genes of signaling pathways: WNT/β-catenin; NF-κB; Notch; Hedgehog; PPAR; p53; TGF-β; STAT/JAK; HIF. The effect of PSMs on signaling pathways was analyzed in the HT29 cell line after 24 h treatment with sanguinarine. This PSM was chosen as acompound with high affinity and distinct effects in the luciferase assay and the PCR experiment. Sanguinarine at a concentration of 1.2 µM negatively affected the expression of 42 genes (more than 2 times) and activated the expression of 6 genes (in the range from 1.5 to 2.4 times). These genes were mainly located in the following clusters: STAT/JAK, Notch, TGFβ, WNT, PPAR, NF-κB, Hedgehog (Figure S3).

## 3. Discussion

G4 structures formed in the promoter regions of eukaryotic genes were shown to modulate their transcription [11]. It is assumed that the effect is based on three mechanisms. The main explanation is that G4s inhibit the movement of RNA polymerase, being a steric obstacle to the enzyme’s pathway. Second, some G4s can prevent or promote transcription factors’ recruitment to regulatory regions of genes. Finally, G4s are thought to recruit G4-binding proteins that repress transcription [39].

In our study, we selected six G-rich DNA fragments derived from the promoter regions of the most studied human oncogenes (Table 1), which showed a high probability of in vitro folding into G4 structures under physiologically relevant conditions. As shown by chemical probing, NMR, and CD spectroscopy, these oligonucleotide models usually fold in K^+^ solutions into parallel-stranded intramolecular quadruplexes [2,26,29], although some publication report conformational heterogeneity of the promoter G4s [40]. For comparison, telomeric DNA repeats were used, which fold into G4s with antiparallel and hybrid (3 + 1) topologies [28]. Compared to telomeres, the G4-forming sequences found in the promoter regions are more diverse, as varying numbers and lengths of G-tracts lead to the potential formation of multiple G4s.

Our study focused on plant secondary metabolites, which possess anticancer activity. According to the literature, some of them have the ability to bind G4 structures selectively. Thus, quercetin stabilizes *c-MYC* and telomeric G4s [17,41,42], sanguinarine stabilizes *c-MYC*, *KRAS*, and telomeric G4s [18,43,44], EGCG binds telomeric G4s [45], kaempferol stabilizes *c-MYC* and *VEGFA* G4s and binds *c-KIT* and telomeric G4s [46,47]; thymoquinone stabilizes telomeric G4 [16]; curcumin is able to stabilize *KRAS* G4 [48].

Here we have increased the number of PSMs analyzed in one model system for their ability to selectively bind to the promoter G4s. G4-FID assay data revealed that out of 11 studied PSMs, 6 compounds bind to G4s formed in the promoter regions of 6 oncogenes, although the binding constants varied depending on the nature of the ligand and the target quadruplex (Figure 1 and Table 2). Among the compounds studied, sanguinarine showed the highest affinity for all G4s used, regardless of their topology. Probably it was observed due to its special chemical structure with a wider planar aromatic system compared to other ligands, which is necessary for stacking interactions with the external G-tetrads [49], the positive charge also enhancing its binding to the negatively charged G4 core [50]. The multi-targeted behavior of sanguinarine and some other PSMs is likely due to the ability of many G4-binding ligands to recognize a predominantly peculiar three-dimensional architecture of the G4 core, which is quasi-globular and more like tertiary protein structures than linear double-stranded B-DNA.

So far, very few small molecule ligands have been reported to exhibit selectivity among the various G4 structures. But our data revealed the selectivity of some PSMs for G4s of certain topologies. Thus, sanguinarine preferentially recognizes the parallel-stranded promoter G4s over the antiparallel G4s adopted by human telomeric repeats, while quercetin slightly prefers G4s with antiparallel strand orientation. The ligand with the lowest G4 affinity—curcumin—was shown to weakly bind only *c-MYC* G4, probably due to the small aromatic systems localized at the ends of the molecule and not forming a wide planar core. It should be noted that data on the binding of *c-MYC* G4 to curcumin, as well as to EGCG and thymoquinone, have not been published; this was demonstrated for the first time in our study.

The 26-nt fragment of the *c-MYC* promoter, which can fold into an intramolecular parallel-stranded G4 structure, was chosen for subsequent experiments because it showed the ability to bind the largest number of PSMs under consideration with relatively high affinity. In addition, the *c-MYC* gene product is known to be a major factor in cancer development. Its overexpression is strongly associated with autonomous proliferation, chromosome translocation, and impaired apoptosis in cancer cells [13,51]. *c-MYC* G4 was used in the FID assay to evaluate the relative binding affinity of the most promising PSM ligands to the G4 and DNA duplex structures. Binding constants for the DNA duplex were significantly lower than those for the promoter G4 for all four ligands (Table 2) and the highest selectivity factors were found for quercetin (selectivity factor of 223.96) and thymoquinone, which does not bind to duplex DNA at all. Structural selectivity is a very important factor, since the ability of small molecule ligands to regulate gene expression depends not only on the dynamic equilibrium between G4 and B-DNA in the cell but also on the relative affinity of the ligands for the quadruplex and duplex targets.

The same *c-MYC* quadruplex model was also applied to evaluate the G4 stabilizing effect of the best G4 binders by the UV-spectroscopy method. An increase in thermal stability of *c-MYC* G4 was observed with the addition of 1 or 2 equivalents of sanguinarine and quercetin, while kaempferol and thymoquinone had no visible effect at a non-physiologically low concentration of potassium ions (0.5 mM). This K^+^ concentration, far from that in the human cells (~150 mM KCl), was chosen to capture the entire conformation transition of the extremely stable polymorphic *c-MYC* G4 in the accessible temperature range. According to CD spectroscopy, the addition of an equimolar concentration of dsDNA to *c-MYC* G4 probes containing 2 equivalents of sanguinarine or quercetin did not reduce the G4 stabilizing effect of these PSMs (Figure 3). The results were interpreted as independent evidence of preferential binding of these ligands to the *c-MYC* G4 structure over duplex targeting, as assessed by the FID assay. The *c-MYC* G4 CD spectrum shows a typical parallel G4 fold; its basic features are not significantly altered by the addition of sanguinarine, quercetin, thymoquinone, and kaempferol, which preferentially recognizes the parallel-stranded promoter G4s (Figure 3B,C and Figure S4).

According to the literature data, the inhibition of *c-MYC* oncogene transcription is usually explained by the stabilization of *c-MYC* G4 induced by small molecule ligands [52], since factors preventing G4 folding, for example, G→A nucleotide substitution in the promoter G4 motif, significantly increase oncogene expression [24,53]. However, in the cellular context, the formation of the G4 structures in promoter regions occurs in competition with the preservation of DNA double helix and is regulated by various oppositely directed driving forces. In our work, the suppressive effect on *c-MYC* promoter activity in malignant HeLa and HT29 cells was proved using a luciferase reporter assay for all 4 studied PSMs. This assay with wild-type and mutant (single G→A substitution) *c-MYC* promoter constructs revealed only G4-mediated transcription regulation, as the mutant G4 motif of the *c-MYC* promoter is unable to form a quadruplex structure. This made it possible to equalize any interfering indirect effects of PSMs on *c-MYC* expression through cell signaling pathways and reveal the only PSM effect induced by *c-MYC* G4-binding. The most active inhibitor was thymoquinone, which reduced luciferase gene expression to 28.7%; lesser changes in the relative expression of reporter gene were observed upon treatment with sanguinarine, quercetin, and kaempferol; these ligands inhibited luciferase expression to 33.6, 45, and 46.3%, respectively. Thus, we demonstrated that all 4 PSMs used induced a G4-mediated decrease in *c-MYC* expression.

Finally, we assessed the ability of these four PSMs to regulate *c-MYC* gene expression in a cellular context using qRT-PCR analysis. mRNA expression profiles were monitored in cancer (HeLa and HT1080) and immortalized (HaCaT and NKE) cell lines. The efficiency of *c-MYC* down-regulation induced by PSMs depended on the cell type and the ligand used (Figure 4). These results are consistent with the data published by Lago et al., who showed that promoter sequences of the same genes in two cell lines had a different G4 folding state depending on the degree of chromatin opening [11,21]. Quercetin and thymoquinone were more active in the HeLa cell line, while the effect of kaempferol and sanguinarine was higher in HT1080 cells. Of note, PSMs did not reduce *c-MYC* expression in the immortalized NKE and HaCaT cells, with the exception of thymoquinone, which mediated a significant decrease in *c-MYC* expression in the HaCaT cell line. The preferential activity of PSM ligands in HeLa and HT1080 cell lines is explained by different expression levels of the *c-MYC* oncogene in cancer and immortalized cells, which is lower in the immortalized lines. The discrepancies in the magnitude of PSM effects observed by luciferase reporter assay and qRT-PCR in HeLa cells (Figure 3 and Figure 4) may be associated with the multi-targeted activity of the ligands and their influence on various signaling pathways, which is differently detected by these two methods. According to our G4-FID assay data for six G4-forming oncogene promoters and telomere G4, PSM ligands are able to interact with various G4s that are formed simultaneously across the whole human genome. This finding is consistent with our previous results that G4 aptamers designed to inhibit a specific target protein can also modulate the activity of other G4-recognizing proteins [54].

To analyze the effect of PSMs on different signal pathways, we performed a PCR array using RT^2^ Profiler^TM^ PCR Array Human Signal Transduction Pathway Finder, which includes primers for 84 target genes of signaling pathways activated in cancer cells, in particular, WNT/β-catenin; NF-κB; Notch; Hedgehog; PPAR; p53; TGF-β; STAT/JAK and HIF. This approach revealed that more than half of the analyzed genes changed their expression after exposure to sanguinarine.

In summary, thymoquinone and quercetin have been shown to be more suitable for suppressing *c-MYC* expression in cancer cells as they inhibited *c-MYC* promoter activity in both Luciferase reporter assay and qRT-PCR analysis. Recently, the effectiveness of quercetin as an inhibitor of *c-MYC* expression in HeLa cells was shown in [17]. Interestingly, it was quercetin and thymoquinone that exhibited the highest structural selectivity for G4s, while sanguinarine proved to be the best G4 binder. In this regard, we suggest that it is structural selectivity, i.e., the predominant affinity of ligands for quadruplex targets compared to duplex ones, that mainly contributes to the observed down-regulation of gene expression in a cellular context.

In the cellular environments, the formation of the G4 structures in promoter regions occurs in competition with the DNA double helix and is regulated by various oppositely directed factors: chromatin opening, negative DNA supercoiling, different transcription enhancers and inhibitors, G4-stabilizing and destabilizing cellular proteins, exclusion of the C-rich strand from the B-DNA–G4 equilibrium due to the formation of a stable i-motif or co-transcriptional R-loop [55]. We hypothesize that the selective ligand binding to G4s may represent an additional factor contributing to G4 sequestration and prolongation of their lifetime in complex cellular environments.

## 4. Materials and Methods

### 4.1. Genetal Materials

All oligodeoxyribonucleotides (Table 1), synthesized via standard phosphoramidite chemistry and purified by high-pressure liquid chromatography by Syntol Co. (Moscow, Russia), were used without further purification. Their strand concentrations were determined spectrophotometrically at 260 nm using molar extinction coefficients derived from the nearest neighbor algorithm.

The highest purity grade available PSMs, TO, and DMSO used in this work were purchased from Aldrich (Burlington, VT, USA).

### 4.2. Fluorescent Indicator Displacement Assay

The concentrations of TO and DNA oligonucleotides were 1 and 0.5 μM, respectively. Previously, G4-forming or DNA duplex-forming oligonucleotides in 10 mM sodium cacodylate, pH 7.3, containing 100 mM KCl, were annealed by heating the corresponding probes at 90 °C for 3 min, followed by slow cooling to room temperature to improve intramolecular folding. Each G4- or DNA duplex-FID assay was performed in a 96-well quartz microplate. A temperature of 18 °C is kept constant inside the microplate reader. The concentrations of the PSM ligands were 0.1, 0.25, 0.5, 1, 2.5, 5, and 10 μM. The last concentration corresponded to a tenfold excess relative to the TO concentration. Each concentration was analyzed in triplicate and each experiment was performed three times. Fluorescence was registered with Spectra Max Plus Microplate Spectrophotometer (Molecular Devices, San Jose, CA, USA).

The binding constants (*K_b_*) were calculated using the following equation:

$$K_{b}=\left(\frac{I\left(0\right)}{I\left(L\right)}-1\right)\times \frac{1+K_{f}F}{L}$$

where *K_b_* and *K_f_* are the PSM- and TO-binding constants, respectively; *L* and *F* are the concentrations of PSM (ligand) and TO (fluorophore), respectively; *I*(0) and *I*(*L*) are TO fluorescence without a PSM ligand and in the presence of a ligand with a concentration of *L* [37,38]. *L* required to reduce TO fluorescence by 50% (DC_50_) was detected from the plots and used to simplify the equation:

$$K_{b}=\frac{1+K_{f}F}{L},$$

TO binding constants for *c-MYC* G4 and G4 formed by human telomeric repeats and DNA duplex were used in accordance with published data: *K_b c-MYC_* = 1.50 (±0.17) × 10^6^ M^−1^; *K_b Tel_* = 2.62 (±0.27) × 10^6^ M^−1^ and *K*_bdu_ = 3.03 (±0.53) × 10^6^ M^−1^, respectively [20]. The binding constants for G4s formed by the promoter regions of other oncogenes (*K_b_*_G4_) were chosen to be 2.5 × 10^6^ M^−1^ (average binding constant of G4s with different topologies) [20].

### 4.3. UV Spectroscopy Melting Curves

DNA oligonucleotides containing the G4 motifs were annealed in 20 mM Tris–HCl buffer, pH 7.3, containing 140 mM NaCl and 5 mM KCl (buffer A), or in 20 mM Tris–HCl buffer, pH 7.3, containing 140 mM NaCl and 0.5 mM KCl (buffer B) to promote G4 formation. One or two equivalents of PSM ligands were added to pre-annealed DNA probes; the mixtures were kept for 30 min at room temperature. The absorbance profiles of DNA samples as a function of temperature (at a concentration of ~3 µM per oligonucleotide strand) were recorded in a 600-μL quartz microcuvette (Hellma Analytics, Müllheim, Germany) with an optical path length of 10 mm on a double-beam Hitachi U-2900 UV/visible spectrophotometer (Tokyo, Japan) equipped with a Hitachi thermoelectric controller. Changes in absorbance were monitored between 20 and 85 °C at 295 nm at a heating rate of 1 °C/min. T_m_, defined as the mid-point temperature, was estimated from the extremum value of the first derivative of the fitted curve for data smoothed with the Savitzky–Golay filter and indicated the M ± SD of three independent experiments.

### 4.4. CD Measurements

The procedure of sample preparation for CD measurements was the same as for UV melting experiments. The CD spectra of DNA oligonucleotides were recorded in buffer B in a quartz cuvette of 10-mm optical path length at room temperature or between 30 and 85 °C in 5 °C increments at an average heating rate of 0.5 °C/min on a Chirascan CD spectrometer (Applied Photophysics Ltd., Leatherhead, UK) equipped with a thermoelectric controller with a Peltier element. Two equivalents of PSM ligands and one equivalent of dsDNA were added to pre-annealed DNA probes. The DNA concentration was chosen to attain absorption of 0.6–0.8 at 260 nm, which gives an optimum signal-to-noise ratio. The measurements were performed in the 220–360 nm wavelength range at a scanning speed of 30 nm/min and a signal averaging time of 2 s with a constant flow of dry nitrogen. All the CD spectra were baseline-corrected for signal contributions caused by the buffer. CD spectra were plotted as molar dichroism per oligonucleotide strand against wavelength. The spectra were processed with the Origin 8.0 software using the Savitzky–Golay filter. The CD melting profiles revealed a temperature dependence of a CD signal at 263 nm. T_m_ values were estimated from an extremum value of the first derivative of the fitted curves.

### 4.5. Cell Cultures

Human cervical cancer cells (HeLa), fibrosarcoma cell line (HT1080), immortalized human keratinocyte cells (HaCaT), and immortalized kidney epithelial cell line (NKE-hTERT) were obtained from the Blokhin CRC cell collection. Cells were cultured in Dulbecco’s Modified Eagle Medium (DMEM) supplemented with L-glutamine (0.584 mg/mL), penicillin (50 U/mL), and streptomycin (50 μg/mL) (PanEco, Moscow, Russia) and 10% fetal bovine serum (PanEco). Cell lines were incubated at 37 °C and 5% CO_2_.

### 4.6. Cell Viability Assay

The maximum nontoxic concentrations of PSMs were measured for different cell lines using the MTT test based on the colorimetric determination of the metabolic activity of viable cells. For analysis, cancer cells (HeLa and HT1080) and cells of immortalized cell lines (NKE-hTERT and HaCaT) were seeded in 96-well plates (10,000 cells per well in 200 μL of DMEM). Cells were treated with PSMs of various concentrations for 48 h. Then, 10 μL of MTT reagent solution (5 mg/mL; 0.9% NaCl) was added to each well and incubated for 4 h at 37 °C. The plates were read on a Multiskan FC spectrophotometer (Thermo Scientific, Waltham, MA, USA) using a test wavelength of 570 nm. IC_50_ and IC_20_ values and nontoxic concentrations were derived from MTT absorbance curves plotted against the logarithm of the PSM concentration. All experiments were carried out in parallel and in triplicate.

### 4.7. Construction of Reporter Luciferase Vectors, Transfection, and Luciferase Assays

The promoter sequence (commonly used as part of the Del4 plasmid [56]) of the *c-MYC* gene with wild-type G4 motif was cloned into a pGL3-Basic firefly luciferase promoterless vector (Promega, Madison, WI, USA) at the KpnI and HindIII restriction sites. The promoter sequence was previously PCR amplified from genomic DNA isolated from the HeLa cell line with the following primers:

forward: 5′-CGACCTGGTACCCAGCTGTTCCGCCTGCGATGATTTATAC-3′,
reverse: 5′-CTCGTCAAGCTTCAGCTGCAAGGAGAGCCTTTC-3′.

The wild-type G4 motif in the reporter plasmid was then subjected to site-directed mutagenesis to induce a G→A substitution in the third G-tract to prevent the G4 formation. The primers for mutagenesis are as follows:

forward: 5′-pGGGGAGGGTGAGGAGGGTGGGGAAGG-3′,
reverse: 5′-ATAAGCGCCCCTCCCGGGTTC-3′.

The PCR product was treated with DpnI enzyme (NEB) to remove the wild-type plasmid. Then the linear PCR product was ligated with T4 DNA ligase (SibEnzyme, Novosibirsk, Russia). The G4 motifs upstream of the luciferase reporter gene in the plasmids used in our study are as follows:

wild-type: 5′-GGGGAGGGTGGGGAGGGTGGGGAAGG-3′,
mutated: 5′-GGGGAGGGTGAGGAGGGTGGGGAAGG-3′ (substitution underlined).

Both wild-type and mutated plasmids as well as pGL4.74[hRluc/TK] plasmid (with Renilla luciferase gene) were transfected into HeLa and HT29 cells in 60 mm dishes using TurboFect Transfection Reagent (ThermoScientific, Waltham, MA, USA) according to the manufacturer’s protocol. After 48 h transfection, cells were seeded in 96-well plates and treated with PSMs for 24 h. Cells were then lysed and a luciferase assay was performed using the Dual-Glo Luciferase Assay System (Promega, USA) according to the manufacturer’s protocol. Firefly luciferase activity was normalized to the Renilla luciferase activity. The ratio of luciferase activity in the wild-type plasmid to that in the mutated plasmid was calculated.

### 4.8. RNA Isolation, cDNA Synthesis, and qRT-PCR Assay

The effect of PSMs on *c-MYC* oncogene expression was evaluated in two cancer cell lines (HeLa and HT1080) and two immortalized cell lines (NKE-hTERT and HaCaT) using the qRT-PCR assay. Cells were incubated for 48 h in the medium with various concentrations of PSMs in 6-well plates. Then, total RNA was extracted with TRIzol reagent (ThermoScientific, USA) according to the manufacturer’s protocol. For cDNA synthesis, total RNA (1 μg from both untreated and PSM-treated cells) was reverse transcribed using MMLV-RT reverse transcriptase and random hexanucleotide primers in 25 μL of the reaction mixture according to the manufacturer’s protocol (Syntol). Real-time qRT-PCR was performed using the following thermal cycling conditions: initial denaturation step by heating at 95 °C for 5 min, followed by 40 cycles of 15 s denaturation (at 95 °C), 20 s at the appropriate temperature (depending on the T_m_ values of the primer used) and 25 s of extension at 72 °C. Expression of the gene of interest was normalized to the constitutively expressed housekeeping genes RPLP0, GPI, and RPL27. The relative expression level was calculated for each sample using the 2^−ΔΔCt^ method.

The sequences of the gene-specific primers used for qRT-PCR were as follows:

*RPL27* (forward): 5′-ACCGTCACCCCCGCAAAGTG-3′,
*RPL27* (reverse): 5′-CCCGTCGGGCCTTGCGTTTA-3′,
*c-MYC* (forward): 5′-GGGAGGCTATTCTGCCCATTT-3′,
*c-MYC* (reverse): 5′-CGTAGTCGAGGTCATAGTTCCTG-3′,
*RPLP0* (forward): 5′-CCTCGTGGAAGTGACATCGT-3′,
*RPLP0* (reverse): 5′-CTGTCTTCCCTGGGCATCAC-3′,
*GPI* (forward): 5′-GGAGACCATCACGAATGCAGA-3′,
*GPI* (reverse): 5′-TAGACAGGGCAACAAAGTGCT-3′.

### 4.9. PCR Array

The influence of sanguinarine on the expression of target genes was evaluated in the HT29 cancer cell line using qRT-PCR analysis. Real-time PCR was conducted in 96-well plates RT^2^ Profiler^TM^ PCR Array Human Signal Transduction PathwayFinder (QIAGEN, Hilden, Germany).

The reaction mixture in each well was prepared according to the manufacturer’s protocol. The thermal cycling conditions were as follows: an initial denaturation step by heating at 95 °C for 10 min, followed by 40 cycles of 15 s initial denaturation (at 95 °C) and 1 min of annealing and extension at 60 °C. The expression of the gene of interest was normalized to the constitutively expressed housekeeping genes (ACTB, B2M, GAPDH, HPRT1, RPLP0). The relative expression level was calculated for each sample using the 2^−ΔΔCt^ method in the manufacturer’s software. All experiments were performed three times and in triplicate.

### 4.10. Statistical Analysis

To assess the significance of differences between the groups, including the expression levels, a paired two-sample Student’s *t*-test with a level of statistical significance of *p* < 0.05 was used. Data processing was carried out using Statistics for Windows software.

## 5. Conclusions

G4-forming sequences observed in oncogene promoters have received a great deal of attention as potential biomedical targets for anticancer therapy. The main goal of our study was to clarify whether plant metabolites that bind to the promoter G4s can influence gene expression in a cellular context. Using FID assay, we revealed that many of the PSMs studied effectively bind to G4s of various oncogenes, showing their multi-targeted activity. The ability of the most promising PSMs to regulate luciferase gene transcription controlled by the *c-MYC* promoter was shown in a coupled reporter assay that contained the wild-type promoter sequence and the same one with a G to A substitution preventing G4 folding. This altered *c-MYC* promoter sequence was used to eliminate interfering non-directed influences of PSM ligands and to determine the targeted G4-mediated effect. According to data obtained by qRT-PCR analysis in cultured cancer and immortalized cells of different types, PSM-induced down-regulation of *c-MYC* expression varies depending on the cell line. Importantly, the effect of PSMs obtained using this approach was less intense compared to that revealed by the luciferase reporter assay. It can be explained by the indirect action of PSMs on cell signaling as a result of their multi-target activity. Our study revealed that more than half of the analyzed genes changed their expression level under the influence of PSMs.

## Figures and Tables

**Table 1:** The DNA sequences of oligonucleotides used in the study.

Sequence Source	Sequence, 5′-3′	Secondary Structure Formed	Reference
*VEGF*	GGGGCGGGCCGGGGGCGGGG	Parallel G4	[29]
*c-MYC*	GGGGAGGGTGGGGAGGGTGGGGAAGG		[30]
*WNT1*	GGGGGCCACCGGGCAGGGGGCGGGGG		[31]
*KRAS*	GGGCGGTGTGGGAAGAGGGAAGAGGGGGAGG		[32]
*BCL2*	AGGGGCGGGCGCGGGAGGAAGGGGGCGGGAGCGGGGC		[33]
*c-KIT*	GGGAGGGCGCTGGGAGGAGGG		[34]
*Tel*	GGGTTAGGGTTAGGGTTAGGGTTA	Antiparallel/hybrid G4	[27]
Artificial	CAATCGGATCGAATTCGATCCGATTG	dsDNA hairpin	[15]

**Table 2:** PSM interaction with various G4s and DNA duplex analyzed by GFID assay.

Sequence	Sanguinarine		Quercetin		EGCG		Kaempferol		Thymoquinone	
	DC_50_ µM	*K_b_* (×10^6^ M^−1^)	DC_50_ µM	*K_b_* (×10^6^ M^−1^)	DC_50_ µM	*K_b_* (×10^6^ M^−1^)	DC_50_ µM	*K_b_* (×10^6^ M^−1^)	DC_50_ µM	*K_b_* (×10^6^ M^−1^)
*c-MYC*	0.32 ± 0.02	7.76 ± 0.53	0.47 ± 0.03	5.33 ± 0.36	0.85 ± 0.06	2.94 ± 0.20	2.16 ± 0.15	1.16 ± 0.08	3.97 ± 0.25	0.63 ± 0.04
*WNT1*	1.24 ± 0.17	2.83 ± 0.40	1.69 ± 0.24	2.07 ± 0.30	1.27 ± 0.18	2.75 ± 0.39	1.16 ± 0.16	3.03 ± 0.43	1.29 ± 0.19	2.71 ± 0.39
*VEGFA*	4.07 ± 0.57	0.86 ± 0.12	1.13 ± 0.16	3.10 ± 0.44	2.00 ± 0.28	1.75 ± 0.25	1.67 ± 0.24	2.09 ± 0.30	2.33 ± 0.33	1.50 ± 0.21
*c-KIT*	1.07 ± 0.15	3.28 ± 0.47	0.92 ± 0.13	3.80 ± 0.54	2.43 ± 0.35	1.44 ± 0.21	2.15 ± 0.30	1.63 ± 0.23	2.17 ± 0.31	1.61 ± 0.23
*BCL2*	0.85 ± 0.12	4.12 ± 0.59	0.63 ± 0.09	5.56 ± 0.79	0.96 ± 0.14	3.65 ± 0.52	2.00 ± 0.28	1.75 ± 0.25	1.22 ± 0.10	2.87 ± 0.41
*KRAS*	3.76 ± 0.53	0.93 ± 0.13	2.03 ± 0.30	1.72 ± 0.25	2.16 ± 0.31	1.62 ± 0.23	2.15 ± 0.31	1.63 ± 0.23	2.30 ± 0.33	1.52 ± 0.22
Tel	8.42 ± 0.59	0.43 ± 0.03	0.49 ± 0.04	7.43 ± 0.55	2.00 ± 0.15	1.81 ± 0.14	2.00 ± 0.15	1.81 ± 0.14	2.15 ± 0.17	1.68 ± 0.13
DNA hairpin	3.08 ± 0.40	1.30 ± 0.17	105.26 ± 11.08	0.038 ± 0.004	NA	NA	114.29 ± 6.53	0.035 ± 0.002	-	0
Selectivity factor (DC_50 DNA hairpin_/DC_50 *c-MYC*_)										
*c-MYC*	9.63 ± 0.19		223.96 ± 0.17		NA		52.91 ± 0.13		Absolute	

## Data Availability

The data presented in this study are available on request from the corresponding author.

## Supplementary Materials

The following supporting information can be downloaded at: https://www.mdpi.com/article/10.3390/ijms23169209/s1.

## Abbreviations

G4, G-quadruplex; PSM, plant secondary metabolites; TmPyP4, (5,10,15,20-Tetrakis-(N-methyl-4-pyridyl)porphine); MTT, (3-(4,5-dimethylthiazol-2-yl)-2,5-diphenyl tetrazolium bromide); G4-FID, G-quadruplex fluorescent indicator displacement.
