# Supplementary material for: Effects of G-Quadruplex-Binding Plant Secondary Metabolites on c-MYC Expression
Source: Int J Mol Sci. 2022 Aug 16;23(16):9209. doi: 10.3390/ijms23169209 (PMC9409388; doi:10.3390/ijms23169209)

Effects of G-quadruplex-binding plant secondary metabolites on c-MYC expression

Roman G. Zenkov [1]*, Kirill I. Kirsanov[1,2], Anna M. Ogloblina[1], Olga A. Vlasova[1], Denis S. Naberezhnov[1], Natalia Y. Karpechenko[1,3], Timur I. Fetisov[1], Ekaterina A. Lesovaya[1,4], Gennady A. Belitsky[1], Nina G. Dolinnaya[5], Marianna G. Yakubovskaya[1]

[1] N. N. Blokhin Russian Cancer Research Center, 24 Kashirskoe shosse, Moscow 115478, Russia;

[2] RUDN University, 6 Miklukho-Maklaya St., Moscow 117198, Russia

[3] Depatment of Medicinal Chemistry and Toxicology, Pirogov Russian National Research Medical University, 1 Ostrovityanova st., Moscow 117997, Russia

[4] I.P. Pavlov Ryazan State Medical University, 9 Vysokovoltnaya st., Ryazan 390026, Russia

[5] Department of Chemistry, Lomonosov Moscow State University, Leninskie Gory 1, 119991 Moscow, Russia

*e-mail: r-zenkov@mail.ru

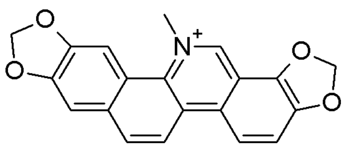

Sanguinarine

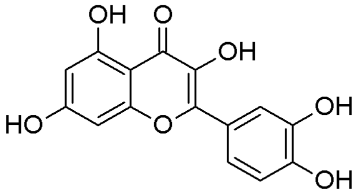

Quercetin

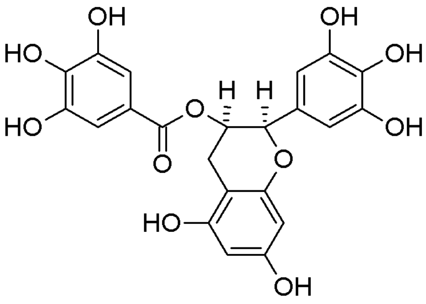

EGCG

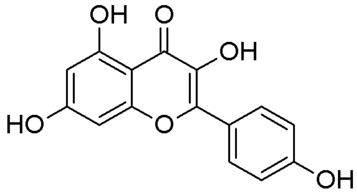

Kaempferol

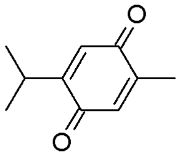

Thymoquinone

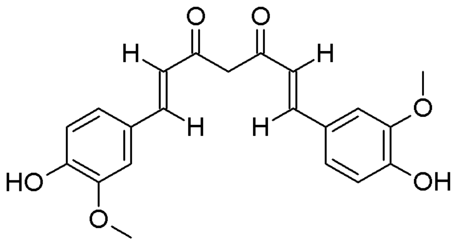

Curcumin

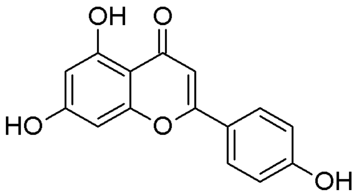

Apigenin

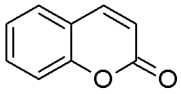

Coumarin

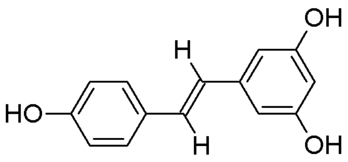

Resveratrol

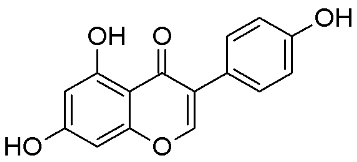

Genistein

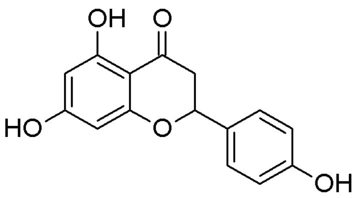

Naringenin

Figure S1. Structural formulae of plant secondary metabolites.

Table S1. IC_{50}, IC_{20} and applied nontoxic concentrations of PSMs in cancer and immortalized cell lines, μM.

Polyphenol	Cell viability	Cell line			
		HeLa	HT1080	NKE-hTERT	HaCaT
Sanguinarine	Applied concentrations	0.16	0.16	0.16	0.16
	IC_{20}	0.54±0.09	0.32±0.04	0.73±0.06	0.54±0.05
	IC_{50}	1.19±0.08	0.69±0.04	2.33±0.19	1.50±0.16
Quercetin	Applied concentrations	8	8	8	8
	IC_{20}	23±12	19±1	31±5	56±3
	IC_{50}	47±16	108±6	109±4	355±76
Kaempferol	Applied concentrations	4	4	32	4
	IC_{20}	14±1	11±1	98±8	13±2
	IC_{50}	73±20	61±1	382±8	54±3
Thymoquinone	Applied concentrations	14	14	14	3.5
	IC_{20}	22±1	20±1	19±1	9±1
	IC_{50}	38±18	35±13	61±3	47±3

Figure S2. Thiazole orange displacement from G4s by PSMs. Fluorescence intensity-concentration curves of all compounds.

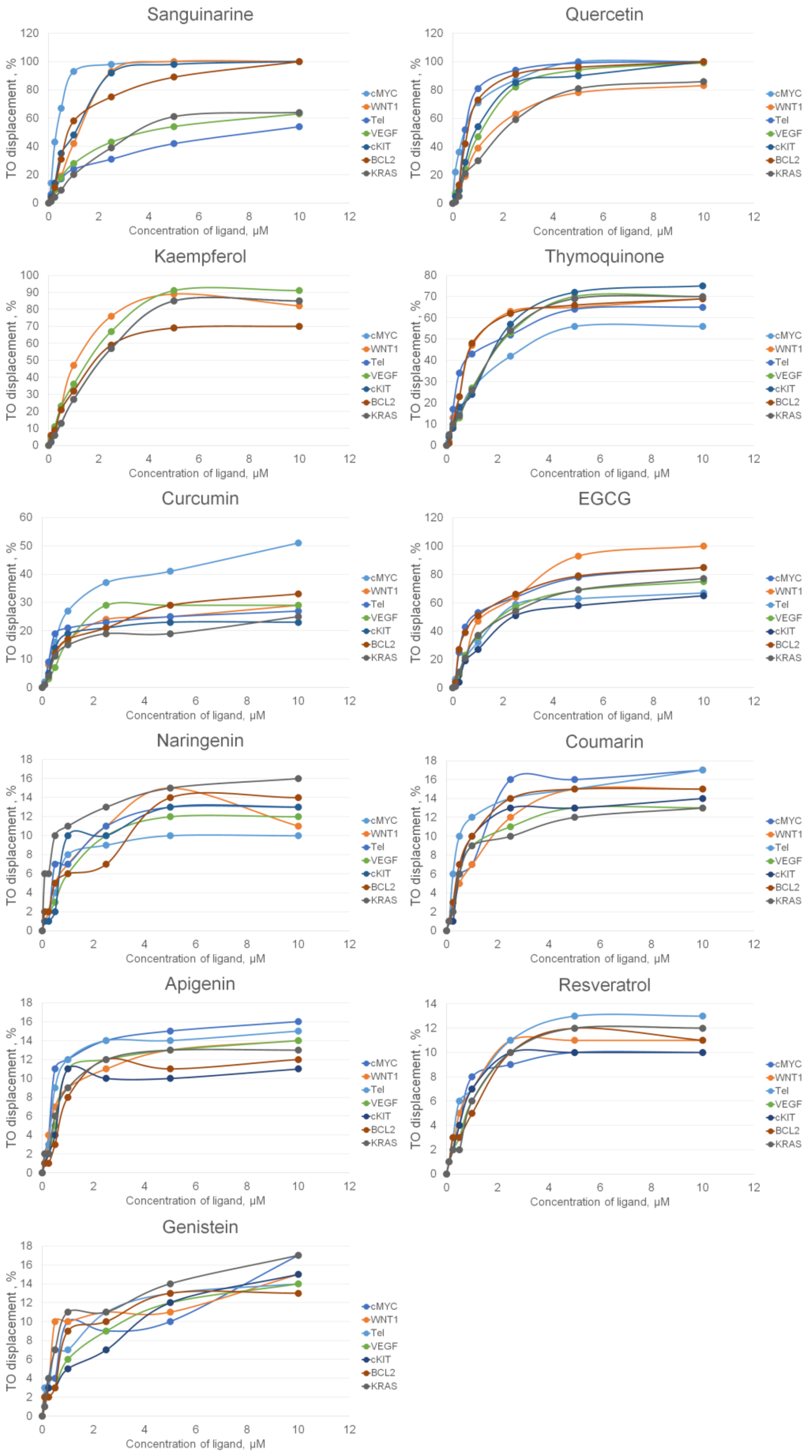

Table S2. Fluorescence data of the PSMs.

Polyphenol	Excitation	Emission	Laser voltage	Influence on the fluorescence of thiazole orange	Fluorescence
EGCG	328 nm	391 nm	900 v	No influence	Low level
Resveratrol	319 nm	400 nm	900 v	No influence	High level
Sanguinarine	480 nm	600 nm	900 v	No influence	High level
Thymoquinone	-	-	-		None
Kaempferol	-	-	-		None
Genistein	-	-	-		None
Quercetin	-	-	-		None
Naringenin	-	-	-		None
Apigenin	-	-	-		None
Coumarin	-	-	-		None
Curcumin	-	-	-		None

Figure S3. Fluorescence spectra of the PSMs.

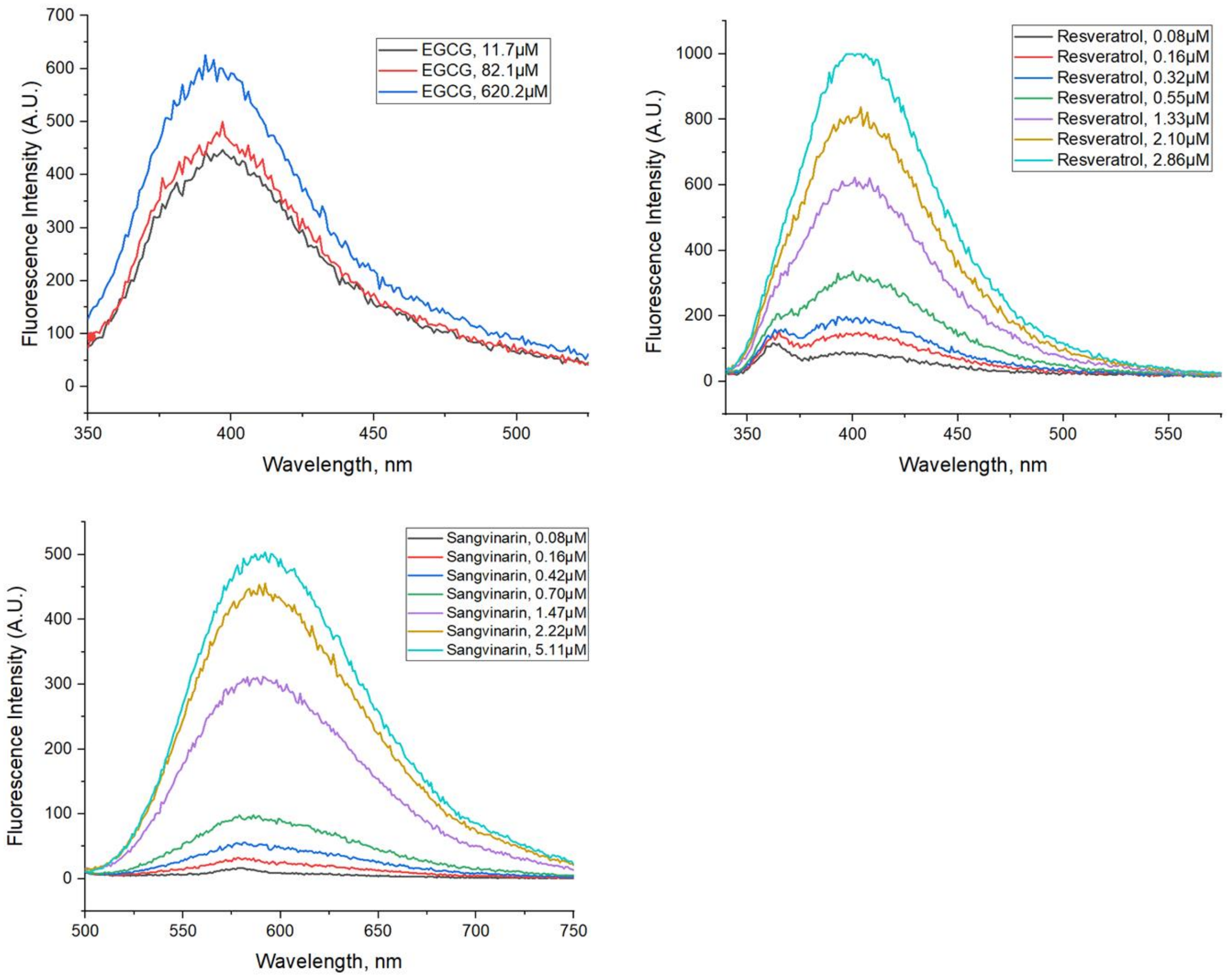

Table S3. Sanguinarine-induced expression changes in a set of target genes of signal pathways. Concentrations of PSMs are presented as µM.

		Compounds	
Signal Pathway	Genes	Sanguinarine 2.5	Sanguinarine 1.2
TGFβ Signaling	ATF4		
	CDKN1B		
	EMP1		
	GADD45B		
	HERPUD1		
	IFRD1		
	MYC		
	TNFSF10		
WNT Signaling	AXIN2		
	CCND1		
	CCND2		
	DAB2		
	FOSL1		
	MMP7		
	MYC		
	PPARD		
	WISP1		
NFκB Signaling	BCL2A1		
	BIRC3		
	CCL5		
	CSF1		
	ICAM1		
	IFNG		
	STAT1		
	TNF		
JAK1 & JAK2 / STAT1-Induced	IRF1		
STAT3-Induced	BCL2L1		
	CCND1		
	CEBPD		
	LRG1		
	MCL1		
	SOCS3		
STAT5-Induced	BCL2L1		
	CCND1		
	SOCS3		
JAK1 & JAK3 / STAT6-Induced	FCER2		
JAK1 & JAK3 / STAT6 Responsive	GATA3		
p53 Signaling	BAX		
	BBC3		
	BTG2		
	CDKN1A		
	EGFR		
	FAS		
	GADD45A		
	PCNA		
	RB1		
Notch Signaling	HES1		
	HES5		
	HEY1		
	HEY2		
	HEYL		
	ID1		
	JAG1		
	LFNG		
	NOTCH1		
Hedgehog Signaling	BCL2		
	BMP2		
	BMP4		
	PTCH1		
	WNT1		
	WNT2B		
	WNT3A		
	WNT5A		
	WNT6		
PPAR Signaling	ACSL3		
	ACSL4		
	ACSL5		
	CPT2		
	FABP1		
	OLR1		
	SLC27A4		
	SOCS3		
Oxidative Stress	FTH1		
	GCLC		
	GCLM		
	GSR		
	HMOX1		
	NQO1		
	SQSTM1		
	TXN		
	TXNRD1		
Hypoxia Signaling	ADM		
	ARNT		
	CA9		
	EPO		
	HMOX1		
	LDHA		
	SERPINE1		
	SLC2A1		
	VEGFA		

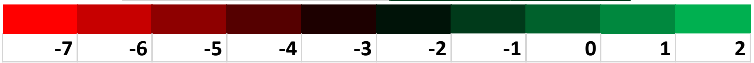

Log_2(fold change)

Figure S4. CD spectra of c-MYC G4 (~4 µM oligonucleotide strand concentration) supplemented with 2 equivalent of kaempferol (A) or thymoquinone (B) and 1 equivalent of dsDNA. The measurements were carried out at room temperatures in buffer B.

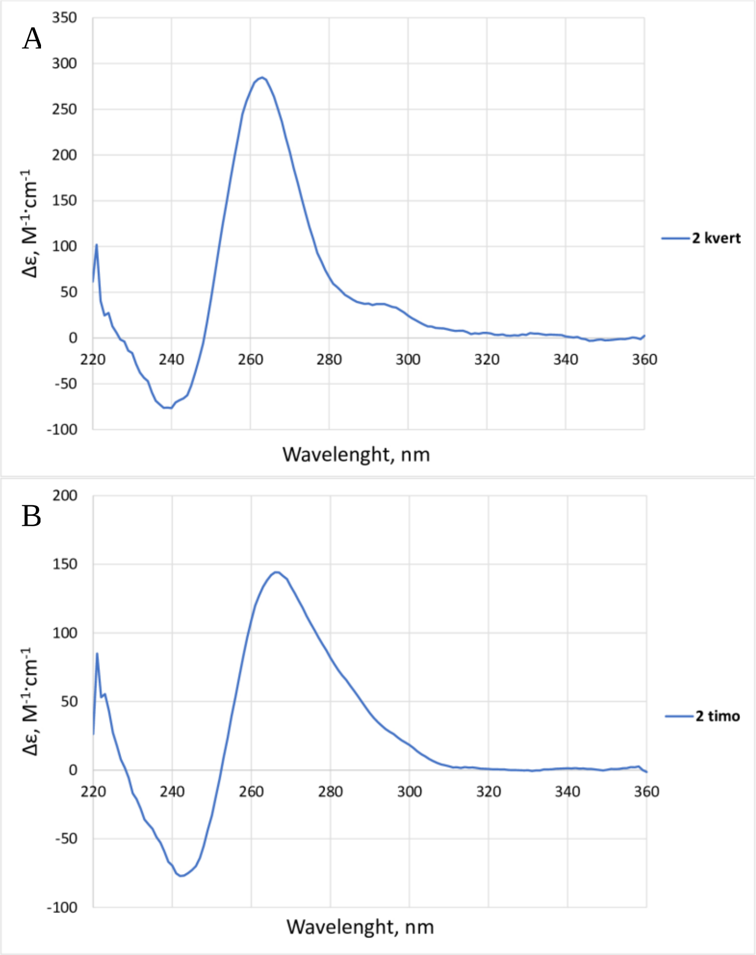

Supplement: Supplementary file 1 [file ijms-23-09209-s001.zip › ijms-1871470-supplementary.pdf]
